# Dental Health Status of Incarcerated Individuals in Silesia: A Five-Year Retrospective Case-Control Study

**DOI:** 10.3390/jcm14165909

**Published:** 2025-08-21

**Authors:** Jakub Fiegler-Rudol, Piotr Ziobro, Anna Zawilska, Karolina Lau, Janusz Kasperczyk

**Affiliations:** 1 Student Scientific Society at the Department of Conservative Dentistry and Endodontics, Faculty of Medical Sciences in Zabrze, Silesian Medical University in Katowice, Plac Akademicki 17, 41-902 Bytom, Poland; s86367@365.sum.edu.pl; 2 Department of Conservative Dentistry and Endodontics, Faculty of Medical Sciences in Zabrze, Silesian Medical University in Katowice, Plac Akademicki 17, 41-902 Bytom, Poland; azawilska@sum.edu.pl; 3 Department of Medicine and Environmental Epidemiology, Faculty of Medical Sciences in Zabrze, Silesian Medical University in Katowice, Jordana 19, 41-808 Zabrze, Poland; karolina.lau@sum.edu.pl (K.L.); jkasperczyk@sum.edu.pl (J.K.)

**Keywords:** case-control studies, dental caries, oral health, oral surgical procedures, prisoners, tooth loss

## Abstract

**Background**: Incarcerated individuals often experience poor oral health due to limited access to care and socioeconomic disadvantages. **Objective**: This study assessed the dental health status of incarcerated individuals in Silesia over a five-year period using the Decayed, Missing, and Filled Teeth (DMFT) index and compared their outcomes to a matched control group from the general population. **Methods**: We conducted a retrospective observational case-control study at the University Centre for Dentistry in Bytom, reviewing records of 136 incarcerated patients (mean age 36.8 ± 7.9 years; 9.4% women) and a matched control group between 2019 and 2024. **Results**: Incarcerated individuals had a higher mean DMFT score (14.4 ± 5.7) compared to controls (11.5 ± 6.5; mean difference = 2.95, 95% CI: 1.53 to 4.37; Cohen’s d = 0.49), with more decayed (4.9 ± 3.2 vs. 3.4 ± 2.4) and missing teeth (4.3 ± 3.2 vs. 3.5 ± 2.6). Most incarcerated patients (65.5%) required oral surgical treatment, most commonly for retained roots (25.9%) and impacted teeth (24.5%). No significant DMFT differences were observed based on age or sex, although disparities were most pronounced in older female prisoners (mean DMFT 17.8 vs. 9.8 in controls aged 40+). **Conclusions**: Incarcerated individuals in Silesia demonstrated a significantly higher burden of untreated dental disease and greater tooth loss compared to non-incarcerated controls over the five-year period. The predominance of advanced dental conditions requiring surgical intervention highlights missed opportunities for early and preventive care in this vulnerable population.

## 1. Introduction

### 1.1. Rationale

Oral health is a fundamental component of general health and quality of life, yet significant disparities persist in access to dental care and disease burden, particularly among vulnerable and marginalized populations [1,2]. Incarcerated individuals are widely recognized as a high-risk group for poor oral health outcomes due to a convergence of socioeconomic disadvantage, high rates of substance use, and chronic barriers to care [3,4,5,6]. Studies from North America and Western Europe have consistently shown that people in correctional facilities experience a higher prevalence of dental caries, tooth loss, and unmet treatment needs compared to the general population [7,8,9]. These disparities are compounded by the unique challenges of the prison environment, including restricted access to dental services, delays in seeking care, and prioritization of urgent or emergency treatment over preventive and restorative approaches [10,11]. International evidence highlights a broad range of oral health problems in prison settings, including advanced untreated caries, high rates of missing teeth, and limited provision of prosthetic or restorative care [12,13]. In the United States, for example, mean DMFT (Decayed, Missing, Filled Teeth) scores among incarcerated adults are substantially higher than in non-incarcerated populations, with many inmates requiring complex surgical intervention [14]. Similar findings have been reported in the United Kingdom, where lengthy wait times and limited access to routine dental examinations are prevalent [15]. Research also suggests that women and older inmates may face even greater oral health challenges, potentially due to compounded vulnerabilities such as prior trauma, poor pre-incarceration health, and inadequate access to care [16,17,18]. Despite the recognition of oral health as a component of the right to health, routine dental care in many correctional systems remains underprioritized and underfunded [19,20,21]. In Central and Eastern Europe, evidence on the dental health of incarcerated populations is limited. Most studies in this area are limited by small sample sizes, short durations, or narrow focus, hindering generalizability and long-term monitoring. While poor oral health among incarcerated populations is well documented internationally, multi-year, comparative studies from Central and Eastern Europe are lacking. There has been no systematic comparison of oral health needs and treatment patterns between incarcerated individuals in Silesia and matched controls from the general population. Existing data are insufficient for targeted policy or evaluating the impact of correctional dental care in this region. Understanding the dental health status of prisoners relative to the general population is essential for informed policy, resource allocation, and preventive strategies. Retrospective studies using standardized indices such as DMFT can provide valuable baseline data, highlight service gaps, and guide targeted interventions.

### 1.2. Objectives

The present study aims to address these gaps by conducting a retrospective case-control analysis of dental health among incarcerated individuals in Silesia over a five-year period. The specific objectives are as follows:

1. To assess and compare the dental health status of incarcerated individuals and matched non-incarcerated controls in Silesia using the DMFT index.
2. To characterize the types and severity of dental conditions among incarcerated patients.
3. To evaluate age-specific and sex-specific patterns in dental health outcomes within and between groups.
4. To identify potential disparities in oral healthcare utilization and treatment needs among prisoners versus controls.

We hypothesize that incarcerated individuals in Silesia have significantly poorer dental health, as measured by higher DMFT indices and a greater prevalence of untreated dental disease, compared to matched non-incarcerated controls from the general population.

## 2. Materials and Methods

### 2.1. Study Design and Setting

This study used a retrospective matched case-control design to compare dental health status between incarcerated individuals and non-incarcerated controls. It was conducted at the University Centre for Dentistry in Bytom. The review covered a five-year period, from 1 January 2019 to 1 January 2024. The study population comprised incarcerated individuals from four local prisons, located in Bytom, Gliwice, Tarnowskie Góry, and Zabrze, who received dental treatment at the institution during the specified timeframe. The University Centre for Dentistry in Bytom provides comprehensive dental care services, including preventive, restorative, endodontic, and surgical treatments. It also serves as a referral centre for specialized dental procedures. Inmates are referred for dental care by prison healthcare providers based on the urgency and type of dental ailment. The prisons in Bytom, Gliwice, Tarnowskie Góry and Zabrze have established referral protocols for dental emergencies, preventive care, and elective procedures, thereby allowing a structured approach for inmates to access dental services.

### 2.2. Sample Size

As this was a retrospective analysis that included all eligible cases identified during the five-year period, no formal sample size calculation or a priori power analysis was performed. The final sample size was determined by the number of incarcerated individuals and matched controls who met the inclusion criteria. While this approach maximized the available data, we acknowledge that it may limit the ability to detect small effect sizes, a limitation addressed further in the discussion section. To evaluate the adequacy of the sample size, a post hoc power analysis was conducted using the observed effect size for the difference in mean DMFT scores between incarcerated and control groups. With 136 individuals in each group, an alpha level of 0.05, and an observed effect size (Cohen’s d) of approximately 0.49, the estimated statistical power (1 − β) was 0.90. This indicates that the study was adequately powered to detect significant group differences in dental health outcomes.

### 2.3. Patient Selection and Data Collection

All patients who were documented as prisoners in their medical records and received dental care between 1 January 2019 and 1 January 2024 were considered for inclusion in this study. Table 1 shows the selection criteria for the study and control group.

### 2.4. Data Retrieval Process

Both paper-based archives and electronic health records were systematically reviewed. Institutional staff with appropriate clearance accessed these records under supervision to maintain data privacy. A list of potential prisoner-patient identifiers was obtained from administrative logs. Subsequently, each patient’s file was examined to confirm incarceration status and retrieve relevant dental health data. Three authors independently reviewed each eligible patient record. They extracted information on demographics (age, sex, and prison facility), dental charting, clinical history, and radiographic findings to ensure completeness and accuracy. Discrepancies were resolved by consultation with a third reviewer.

### 2.5. Inter-Reviewer Reliability

To ensure inter-reviewer reliability in DMFT scoring, all authors participated in a standardized training session and used a shared reference manual. A pilot reliability assessment was performed on a random subset of patient records, with discrepancies resolved through consensus. Throughout the study, 10% of records were independently reviewed by a second reviewer, and inter-rater agreement was monitored using Cohen’s kappa coefficient (kappa < 0.80). Once extracted, the dataset was checked for completeness and consistency. Records with missing crucial information (e.g., patient ID or DMFT components) were either corrected if verifiable data were found or excluded if the missing data could not be reliably retrieved.

### 2.6. DMFT Index

DMFT scores were extracted from clinical records from the data obtained by dentists during clinical visits. The DMFT score consists of a sum of Decayed (D), Missing (M), and Filled (F) teeth [22]. Decayed Teeth (D): Cavitated lesions or radiographic evidence indicating active caries were recorded. Missing Teeth (M): Teeth extracted or absent due to caries or other dental pathologies (excluding congenital absence) were counted. Filled Teeth (F): Teeth restored with amalgam, composite, or other restorative materials were noted. Where applicable, the interpretation of panoramic or periapical X-rays aided in confirming diagnoses (e.g., carious lesions, retained roots, or impacted teeth). A digital data collection spreadsheet was used to record and standardize information. Data were then entered into a secure database, with anonymized patient IDs replacing any personally identifiable information. Consistency checks and validation rules (e.g., checks for DMFT values) were applied to minimize data entry errors.

### 2.7. Department of Admission and Primary Diagnosis

Based on the patient’s primary treatment needs and referral details, each case was classified into one of two departmental categories: the Restorative Dentistry Department, which included patients requiring preventive procedures, restorative treatments such as fillings and endodontics, or non-surgical management of dental conditions; and the Oral Surgery Department, which encompassed patients presenting with more complex oral or maxillofacial conditions necessitating surgical interventions such as tooth extractions, management of impacted teeth, or removal of retained roots. Each patient’s primary diagnosis and other relevant dental conditions were mapped to the International Classification of Diseases (ICD-10) codes as documented in clinical records. Additionally, the most frequent reasons for seeking dental care, such as acute pain, chronic infection, routine check-ups, or specialist referrals, were categorized to provide insight into patterns of dental morbidity within the incarcerated population.

### 2.8. Control Group

The control group was selected using a manual matching procedure in which each incarcerated patient was matched with a non-incarcerated patient based on age (within ±3 years), sex, and the primary type of dental treatment received (restorative or surgical). Matching was conducted by two independent investigators to reduce selection bias, with any discrepancies resolved through consensus. Table 1 compares the selection criteria of the study group to those of the control group. Nevertheless, we acknowledge that manual matching may still introduce selection bias, as some variables (such as socioeconomic status or comorbidities) could not be accounted for using available records. The control group consisted of 136 non-incarcerated individuals treated at the same University Centre for Dentistry in Bytom during the study period (1 January 2019 to 1 January 2024). Additionally, all control patients resided within the Silesian Voivodeship (Poland) to account for regional variations in healthcare access and socioeconomic conditions. Cases lacking sufficient data or an appropriate match were excluded from the final analysis. The inclusion criteria for the control group mirrored those used for the prisoner cohort, including the availability of complete dental records sufficient for DMFT index calculation. Patients were excluded if they had incomplete documentation or if their records suggested previous incarceration. This methodological alignment allowed for robust, department-matched analysis of dental health outcomes between the two groups, minimizing potential bias related to treatment variability or demographic differences.

### 2.9. Statistical Analysis

All statistical analyses were performed using Statistica software (version 13.3, StatSoft Inc., Tulsa, OK, USA). The primary outcome, DMFT, is a count variable and frequently exhibits non-normal distribution in population studies. In this study, the distribution of DMFT scores for both groups was assessed visually using histograms. Although mild deviations from normality were observed, the sample size was sufficiently large for the central limit theorem to apply, which supports the use of ANOVA for comparing mean DMFT values between groups. To ensure the robustness of our findings, we also performed non-parametric analyses using the Mann–Whitney U test for group comparisons. The results of both the parametric and non-parametric tests were consistent. The methodological rationale for this dual approach is discussed further in the limitations section. Statistical significance was set at *p* < 0.05. For comparisons of oral health indicators between prisoner and control groups within each clinical department and by age–sex strata, *p*-values were calculated using Welch’s independent two-sample *t*-test based on summary statistics (mean, standard deviation, and sample size) for each group. This method accounts for potential inequality of variances between groups and is appropriate when raw patient-level data are unavailable.

### 2.10. Ethical Considerations

This study was conducted in accordance with institutional and national requirements for retrospective chart reviews [23,24]. Ethical approval was granted by the University Centre for Dentistry in Bytom, following internal procedures for retrospective research; however, as the study did not include the direct handling of patients, a formal Institutional Review Board was not required. Approval was provided by the President of the University Centre for Dentistry in accordance with Polish regulations governing the use of de-identified medical records for research purposes. To ensure data confidentiality, all patient identifiers, including names, national identification numbers, and birth dates, were removed before analysis. Each record was assigned a unique, study-specific code, and a master list linking codes to identifiers was kept separately in an encrypted file accessible only to a designated data manager. The main working database, containing only anonymized data, was stored on a password-protected institutional server with access restricted to authorized members of the study team. Data handling, storage, and processing procedures complied fully with the General Data Protection Regulation (GDPR, EU 2016/679) and relevant Polish data protection law [25,26]. All data will be destroyed or permanently anonymized after the completion of the study in accordance with institutional policy.

## 3. Results

### 3.1. General Results

Over the five-year study period (2019–2024), a total of 136 incarcerated individuals received dental treatment at the University Centre for Dentistry in Bytom of the Medical University of Silesia in Katowice. Of these patients, 13 (9.4%) were women, reflecting a smaller but significant female subgroup within the incarcerated population seeking oral healthcare. The patients came from four major correctional facilities across the Silesian region: Tarnowskie Góry (52 individuals; 39.6%), Bytom (50; 36.0%), Gliwice (20; 14.4%), and Zabrze (14; 10.0%). The mean age of the cohort was 36.76 ± 7.88 years, with a median age of 37.50 years, indicating that most patients were in middle adulthood, a demographic known to experience elevated cumulative oral health burdens. Dental status was assessed using the DMFT index, which revealed a significant prevalence of oral disease. The mean DMFT score was significantly higher in prisoners (mean difference = 2.95, 95% CI: 1.53 to 4.37; Cohen’s d = 0.49), indicating a moderate effect size for the association between incarceration and dental disease. The mean DMFT score among incarcerated patients was 14.42 ± 5.74, comprising a mean of 4.91 ± 3.15 decayed teeth, 4.27 ± 3.16 missing teeth, and 5.26 ± 3.97 filled teeth, underscoring a notable combination of active disease, historical tooth loss, and restorative interventions. When compared to the control group, non-incarcerated patients treated at the same center, the control cohort exhibited a lower overall mean DMFT score of 11.47 ± 6.47, with fewer decayed (3.36 ± 2.38) and missing teeth (3.46 ± 2.59), and a slightly lower number of filled teeth (4.65 ± 3.74). Further stratification by clinical department showed that 46 incarcerated patients (33.1%) were treated in the Restorative Dentistry department, while the majority, 91 individuals (65.5%), required care in the Oral Surgery department. Surgical cases predominantly involved tooth extractions, impacted teeth, or retained roots. This perhaps suggests that opportunities for conservative, preventive, or early restorative treatment were frequently missed or unavailable. The main characteristics of patients are summarized in Table 2.

### 3.2. Group Comparisons

To further assess dental status, incarcerated patients were compared to non-incarcerated controls matched by clinical department. Prisoners in the oral surgery group had significantly more missing teeth (*p* = 0.04) and higher DMFT scores (*p* = 0.03) than controls, with decayed teeth showing a borderline difference (*p* = 0.05). In restorative dentistry, no statistically significant differences were found between the prisoner and control groups for any oral health indicators (Table 3).

Stratified analyses revealed that male prisoners had consistently higher mean DMFT scores compared to male controls across all age groups, with the largest difference observed in the 30–39 and 40+ categories (mean difference: 3.9 and 3.0 DMFT, respectively). Among females, the difference between prisoners and controls was most notable in the 40+ group (mean DMFT: 17.8 vs. 9.8). These results suggest that the disparity in dental health associated with incarceration is especially pronounced among older adults and male inmates. The greater differences in DMFT scores among older and male prisoners may reflect the cumulative effects of inadequate access to dental care and longer exposure to risk factors over time. The particularly greater DMFT scores observed in older female inmates, though based on small numbers, warrants further investigation and may signal unique vulnerabilities in this subgroup.

Among individuals under 30, the mean DMFT score was 11.3 ± 2.4 for control females and 11.7 ± 2.7 for incarcerated females. For males in this age group, the scores were 13.4 ± 3.1 in the control group and 14.1 ± 3.3 in the prisoner group. In the 30–39 age group, control females had a mean DMFT of 13.3 ± 2.8, compared to 15.0 ± 2.9 among female prisoners. Control males in this range had a mean of 10.1 ± 2.5, while male prisoners had a higher mean of 14.0 ± 3.0. For those aged 40 and above, female controls had a mean DMFT of 9.8 ± 2.2, which was substantially lower than the 17.8 ± 3.5 observed among female prisoners. Among males in the oldest age group, the mean DMFT was 12.2 ± 2.9 in the control group and 15.2 ± 3.1 among prisoners. The sample size for each group was matched between controls and prisoners within each age and sex category. In the 40+ age group, female prisoners had a mean DMFT 8.0 points higher than female controls (17.8 vs. 9.8; 95% CI: 2.0 to 14.0), whereas among males in the same age group, the difference was 3.0 points (15.2 vs. 12.2; 95% CI: 0.5 to 5.5). Statistically significant differences in DMFT scores were observed among males aged 30–39 (*p* < 0.001) and females aged 40+ (*p* = 0.01), with prisoners having notably higher values than controls. The difference among males aged 40+ approached significance (*p* = 0.06). Other age–sex categories showed no significant differences (*p* ≥ 0.20), suggesting that the largest disparities in dental health are concentrated in middle-aged men and older women.

### 3.3. DMFT Component Percentages and Dental Treatment Index

To further characterize dental health differences, we calculated the percentage contribution of each component to the total DMFT score for both incarcerated and control groups based on per-patient averages. Among incarcerated individuals, decayed teeth accounted for 43.8% of the total DMFT, missing teeth 32.8%, and filled teeth 30.6%. In the control group, decayed teeth represented 31.4%, missing teeth 30.1%, and filled teeth 39.0%. Statistical testing showed that the differences in the proportion of decayed teeth (*p* = 0.051) and filled teeth (*p* = 0.057) approached significance, suggesting a trend toward a higher proportion of untreated decay and a lower proportion of restorative work among prisoners. The proportions of missing teeth did not differ significantly between groups (*p* = 0.570). The Dental Treatment Index (TI), defined as (Filled + Missing)/(Decayed + Missing + Filled) × 100, averaged 63.4% for the incarcerated group and 69.1% for controls, with no statistically significant difference (*p* = 0.270). These results, summarized in Table 4, indicate that prisoners not only bear a higher relative burden of untreated decay, but are also less likely to have received restorative care, reflecting ongoing disparities in access to timely treatment and preventive interventions (Table 5).

### 3.4. Dental Treatment Needs Assessment

To evaluate unmet dental treatment needs, we analyzed the proportion of individuals with at least one untreated decayed tooth (DT > 0) and at least one missing tooth (MT > 0) in each group. Among incarcerated individuals, 94.9% (129 of 136) had at least one untreated decayed tooth, compared to 84.6% (115 of 136) in the control group (*p* = 0.009). Similarly, 95.6% (130 of 136) of prisoners had at least one missing tooth, versus 80.1% (109 of 136) among controls (*p* < 0.001). The need for restorative treatment, defined as the presence of untreated decay requiring fillings, was present in 94.9% (129 of 136) of prisoners and 84.6% (115 of 136) of controls (*p* = 0.009). These findings indicate that nearly all incarcerated individuals in the study had active dental treatment needs, with a substantially higher proportion compared to the general population controls. This highlights an urgent demand for restorative and surgical interventions among prisoners, which is critical information for resource allocation and planning within correctional dental services. The results are summarized in Table 6.

### 3.5. Primary Diagnoses and ICD-10 Classification

Primary dental diagnoses were classified according to the ICD-10 system for both incarcerated individuals and controls. Among incarcerated patients, the most frequent diagnoses were retained tooth roots (K08.3, 26.3%), impacted teeth (K01.1, 24.8%), pulpitis (K04.4, 16.8%), apical periodontitis (K04.1, 16.1%), and unspecified dental caries (K02.9, 11.7%). In the control group, the most common diagnoses were impacted teeth (K01.1, 28.5%) and retained tooth roots (K08.3, 12.4%). Notably, conditions such as pulpitis, apical periodontitis, and unspecified dental caries, which were prevalent among incarcerated patients, were not observed among controls. These differences highlight a greater burden of advanced and untreated dental disease in the incarcerated population compared to controls.

### 3.6. Summary of Key Findings

Incarcerated individuals demonstrated a notably high burden of dental disease, primarily driven by untreated caries and significant tooth loss. Most of these patients were treated in the Oral Surgery department, indicating the prevalence of advanced dental conditions that often necessitated surgical rather than restorative interventions. The average age of the incarcerated cohort was 36.76 years, with most individuals falling into the middle-adulthood age range. When compared to the non-incarcerated control group, prisoners consistently exhibited poorer oral health outcomes across both treatment departments. These findings highlight the urgent need for targeted preventive strategies, early-stage interventions, and the implementation of comprehensive oral healthcare services within correctional facilities.

### 3.7. Analysis of Results

When analyzed across the entire study population without stratification by clinical department, ANOVAs revealed a statistically significant difference in DMFT scores be-tween incarcerated and non-incarcerated individuals (F = 15.93, *p* = 0.000085). The analysis showed that group status (prisoner vs. control) accounted for a meaningful proportion of the variation in dental health, with prisoners exhibiting significantly higher DMFT scores, including higher values for each of the components of the index than the control group. This finding confirms that incarceration is strongly associated with a greater burden of dental disease, reinforcing the need for focused oral health interventions and preventive care strategies within the prison system. Among incarcerated patients treated within the Restorative Dentistry department, ANOVAs showed no statistically significant differences in DMFT scores based on departmental assignment (F = 0.047, *p* = 0.8297). The sum of squares for the effect was minimal (SS = 1.83) relative to the error term (SS = 1715.83), indicating that whether a prisoner was treated in the Restorative versus Surgical department had no meaningful impact on their DMFT scores. This suggests that the burden of dental disease among prisoners remained consistently high, regardless of the type of treatment setting. There were no statistically significant differences in DMFT scores based on age or sex, indicating that these variables did not influence dental health outcomes within the study population. In the multivariate analysis, incarceration status remained an independent and statistically significant predictor of higher DMFT scores (β = 2.92, 95% CI: 1.44 to 4.39, *p* < 0.001), after adjusting for age, sex, and department. Neither age, sex, nor department were significantly associated with DMFT score in the adjusted model (*p* > 0.05 for each variable). This confirmed the independent association between incarceration and higher DMFT scores. This relationship persisted after adjusting for demographic factors and clinical department, reinforcing the need for targeted interventions to address the dental health disparities observed in the incarcerated population.

## 4. Discussion

### 4.1. Comparison with Other Studies

The oral health of incarcerated individuals constitutes a complex and persistent public health concern, as demonstrated by this five-year retrospective study of correctional systems in Silesia [6]. The observed elevation in DMFT scores among prisoners, compared to non-incarcerated controls, reflects a broader global pattern of disproportionate oral disease burden in correctional populations [19,27,28]. In the present cohort, mean DMFT scores surpassed those reported in several international prison settings, such as those documented for life-sentenced inmates in Karnataka, India [12], and the range of 12.9 to 22.1 observed in U.S. federal facilities depending on age group [29]. These elevated scores align with data indicating that incarcerated persons experience consistently higher levels of untreated caries, tooth loss, and retained roots compared to community counterparts [6,22]. Similar findings have been reported internationally. For example, studies from Rikers Island indicated that nearly one-third of female inmates reported oral pain and averaged 10 decayed, missing, or filled teeth [27]. Data on periodontal health also reveal extensive unmet needs, with some studies estimating that nearly 98% of inmates require oral hygiene instruction and approximately half need complex periodontal treatment [28]. These patterns are not solely attributable to individual neglect but can be traced to persistent structural barriers [29]. Within prisons, dental care frequently suffers from underfunding and staff shortages, with care models often prioritizing pain management and extractions rather than preventive or restorative approaches [9,30]. In several countries, including the United States, incarcerated individuals are formally entitled to healthcare under constitutional provisions, yet dental services may be limited in scope, typically restricted to basic exams, extractions, and temporary restorations, and often falling short of recommended clinical standards [3,31,32,33]. Reports from the United Kingdom demonstrate substantial waiting periods, with over 46% of inmates waiting 6 to 12 weeks for basic dental examinations, and 8% experiencing delays greater than 10 weeks before starting treatment [28,34]. Additional logistical barriers, such as administrative delays, security protocols, and communication challenges between prison and dental staff, can further limit access and contribute to prolonged untreated disease [35,36,37,38,39]. Budget constraints may also drive correctional facilities to favor extractions over more resource-intensive options like crowns or endodontic therapy, leading to permanent oral deficits [40,41,42]. Demographic characteristics further contribute to the oral health status of prison populations. Research indicates that women in custody experience particularly high rates of dental disease, which may be linked to increased exposure to poverty, trauma, and inadequate access to healthcare prior to incarceration [4]. Other studies have identified higher rates of edentulism, periodontal disease, and oral pain among incarcerated women, especially those from marginalized ethnic groups [5,23]. Pregnancy represents a distinct period of risk, with poor maternal oral health associated with adverse outcomes for both mother and child [22]. The duration of incarceration also appears to be an important factor; prolonged custody increases the likelihood of untreated decay, with nearly 98% of long-term inmates exhibiting at least one carious lesion [17]. Despite the controlled environment of prisons, ongoing obstacles to oral hygiene persist, including inconsistent access to dental hygiene products, suboptimal dietary offerings, and widespread tobacco and substance use [21,34]. Psychological factors, such as stress associated with incarceration, may further reduce adherence to personal hygiene practices [31]. Post-release, many former inmates continue to experience high rates of dental disease and barriers to accessing care, including financial limitations and lack of insurance coverage [32]. Social stigma related to visible dental problems, such as missing teeth, can have further negative consequences, affecting self-esteem, employment opportunities, and reintegration into society. Qualitative studies have described how poor dental appearance may serve as a barrier to securing stable housing or employment [2]. Data from the United States suggest that formerly incarcerated individuals often experience “moderate declining” trajectories in dental care, characterized by minimal access to treatment after release, thus exacerbating health disparities [14]. Chronic untreated dental pain may also be a significant risk factor for relapse in individuals with a history of substance use, as self-medication is a common coping strategy [19]. There is evidence, however, that targeted interventions can mitigate some of these risks. Structured oral health education initiatives implemented in correctional facilities in India and Norway have resulted in improved knowledge and oral hygiene practices among inmates [8,13]. Interventions such as motivational interviewing and the distribution of dental hygiene kits have also been associated with increased engagement in self-care [15]. These approaches, if adapted to the Polish correctional system, could help address preventable forms of oral disease. Specialized dental care models that are sensitive to gender and trauma histories are also needed to meet the distinct needs of female inmates [4]. In addition, coordinated systems for post-release dental care may help bridge gaps in access, reducing the impact of cost and social stigma [7,14]. Reintegration programs that incorporate oral health screening, preventive services, and patient education have the potential to support more equitable health outcomes following incarceration [33]. The findings from the present study, when considered alongside regional and international evidence, indicate that dental disease in Silesian prisons is shaped by both individual and systemic determinants. These results reinforce the importance of sustained, system-level improvements in correctional dental care infrastructure, including routine screening, appropriate staffing, equitable access to comprehensive treatment, and the integration of oral health into broader correctional health policies [2,6]. Without such measures, the elevated burden of dental disease among incarcerated populations is likely to persist, with implications for both immediate clinical outcomes and longer-term social reintegration [19].

Previous research has suggested potential associations between poor oral health among formerly incarcerated individuals and negative social outcomes, such as difficulties in securing employment, housing, or stable relationships. Some studies also describe a possible relationship between unmanaged dental pain and relapse into substance use, potentially perpetuating cycles of disadvantage and recidivism. However, it is important to note that these links are primarily based on observational and qualitative data, and robust causal evidence is lacking. While these associations are plausible, they may be confounded by a range of underlying social, economic, and behavioral factors that affect both oral health and social outcomes. As such, the present findings should be interpreted within the context of these broader determinants, and causal inferences regarding the impact of oral health on recidivism or substance use cannot be drawn from the available evidence. Similarly, while the present study and others have reported that poor dental appearance and untreated oral disease can negatively influence self-esteem, social acceptance, and employability, these outcomes likely result from a complex interplay of factors beyond oral health status alone. Future research employing longitudinal and intervention designs will be necessary to better delineate the direct and indirect effects of oral health on recidivism, substance use relapse, and broader measures of social reintegration.

### 4.2. Limitations of the Evidence

This study has several important limitations. The retrospective, single-center design may not capture the full range of oral health needs among incarcerated individuals in Silesia or elsewhere, and inclusion was limited to those who sought or were referred for dental treatment, introducing potential selection bias. Data abstracted from multi-provider records over five years may be subject to inconsistent documentation and coding errors, despite efforts to ensure inter-rater reliability. A major limitation is the absence of baseline oral health data prior to incarceration and information on the duration of imprisonment, which restricts the ability to assess the direct effects of incarceration on oral health. The lack of data on socioeconomic status, substance use, and prior dental history further limits adjustment for important confounders. Only a limited set of variables was included in the multivariate models, and the use of parametric methods for count-based outcomes such as DMFT may not fully address the data’s distributional properties. Regional heterogeneity in prison healthcare access and population characteristics across Poland and Europe may also limit the generalizability of these findings. Future studies should employ multicenter designs, standardized data collection, and broader variable inclusion, with particular attention to prospective collection of pre-incarceration oral health status information.

### 4.3. Limitations of This Study

This study has several important limitations. Sampling bias may have affected our findings, as the retrospective single-center design only included individuals who sought or were referred for dental care at a university-affiliated facility, possibly excluding those with unmet or unrecorded needs. Data quality and consistency are a concern, since information was abstracted from clinical records maintained by multiple providers over five years, which may have resulted in inconsistent documentation, diagnostic coding errors, or missing data despite an inter-rater reliability protocol. Generalizability is limited, as the study population may not represent the wider incarcerated population in Silesia, other regions of Poland, or Central and Eastern Europe, where access to dental care and population characteristics may differ. Unmeasured confounding variables remain a challenge; the absence of data on socioeconomic status, pre-incarceration oral health, substance use, or duration of incarceration restricts the interpretation of observed associations. In particular, we did not have information on the length of incarceration for individual participants, which limited our ability to explore potential associations between time served and oral health status. Finally, analytical constraints exist, as only a limited number of variables could be included in the multivariate analysis, and alternative statistical approaches more suitable for count data, such as Poisson or non-parametric regression, were not performed. Addressing these issues in future multicenter, prospective studies will be important for strengthening the evidence base on oral health in correctional settings.

### 4.4. Implications

The findings of this study suggest several policy considerations for correctional oral healthcare in Silesia and potentially comparable settings. The consistently higher burden of dental disease among incarcerated individuals, relative to non-incarcerated controls, indicates a need to review and possibly enhance the availability and scope of dental services within correctional facilities. Targeted preventive strategies, such as regular oral health screenings and education programs, may help reduce disease progression and the demand for surgical interventions. Given the predominance of advanced dental pathology in this population, improving access to timely restorative and preventive care could contribute to better oral health outcomes. However, these recommendations should be contextualized within broader resource constraints and the heterogeneity of prison health systems across Poland and Europe. Future policy development may benefit from multicenter data, standardized care protocols, and further research into the cost-effectiveness and long-term impact of different models of dental care delivery in correctional environments. Collaboration between healthcare providers, correctional administrators, and policymakers will be important to address disparities and to optimize oral health strategies for incarcerated populations.

## 5. Conclusions

This five-year retrospective case-control study provides additional evidence of the high burden of dental disease among incarcerated individuals in Silesia, with significantly higher DMFT scores and a greater need for surgical intervention compared to matched non-incarcerated controls. These results indicate persistent disparities in oral health status that warrant further attention in correctional health services. However, the study’s single-center, retrospective design and the lack of baseline and behavioral data limit the ability to draw broad or causal inferences. The findings should therefore be interpreted as representative only of the specific population studied and not generalized to all incarcerated populations in Poland or beyond. Further multicenter, prospective studies are needed to clarify the determinants and progression of oral disease in correctional settings and to inform the development of targeted preventive and treatment strategies.

## Figures and Tables

**Table 1:** The inclusion and exclusion criteria for incarcerated patients and the control group analyzed in this study.

Criteria	Incarcerated Group	Control Group
Inclusion Criteria	Adults (≥18 years)	Adults (≥18 years)
	Legally incarcerated at time of dental care	No history of incarceration
	Treated at University Centre for Dentistry, Bytom, 1 January 2019–1 January 2024	Treated at University Centre for Dentistry, Bytom, 1 January 2019–1 January 2024
	Dental records sufficient for DMFT calculation (including clinical and, if needed, radiographic documentation)	Dental records sufficient for DMFT calculation (including clinical and, if needed, radiographic documentation)
	Complete demographic data (age, sex, and facility)	Residency in Silesian Voivodeship
		Matched to incarcerated group by age (±3 years), sex, and primary treatment type (restorative or surgical)
Exclusion Criteria	Not incarcerated at time of dental care	Any history or indication of prior incarceration
	Incomplete/missing records preventing DMFT calculation	Incomplete/missing records preventing DMFT calculation
	Duplicate entries (only the most comprehensive included)	Lack of suitable match based on age, sex, or treatment type
	Age under 18 years	Duplicate entries

**Table 2:** Demographic and clinical characteristics of incarcerated patients (2019–2024).

Characteristic	Value
Total number of incarcerated patients	136
Female patients, ***n*** (%)	13 (9.4%)
Correctional facility of origin, ***n*** (%)	
Tarnowskie Góry	52 (39.6%)
Bytom	50 (36.0%)
Gliwice	20 (14.4%)
Zabrze	14 (10.0%)
Mean age, years ± SD	36.76 ± 7.88
Median age, years	37.50
Department treated in, ***n*** (%)	
Restorative dentistry	46 (33.1%)
Oral surgery	91 (65.5%)
Mean DMFT score ± SD (prisoners)	14.42 ± 5.74
Mean decayed teeth ± SD (prisoners)	4.91 ± 3.15
Mean missing teeth ± SD (prisoners)	4.27 ± 3.16
Mean filled teeth ± SD (prisoners)	5.26 ± 3.97
Mean DMFT score ± SD (control)	11.47 ± 6.47
Mean decayed teeth ± SD (control)	3.36 ± 2.38
Mean missing teeth ± SD (control)	3.46 ± 2.59
Mean filled teeth ± SD (control)	4.65 ± 3.74
Mean difference DMFT (95% CI)	2.95 (1.53 to 4.37)
Cohen’s d (effect size for DMFT difference)	0.49

**Table 3:** Comparison of oral health indicators between control and prisoner groups in restorative dentistry and oral surgery.

Department	Variable	Control Mean	Control SD	Prisoner Mean	Prisoner SD	*p*-Value
Restorative Dentistry	Decayed	3.48	2.28	4.87	3.82	0.14
	Missing	3.61	2.65	3.96	3.51	0.71
	Filled	3.83	2.99	4.30	4.27	0.66
	DMFT	10.91	6.18	13.59	5.56	0.13
	Age	36.63	11.11	36.80	7.86	0.95
Oral Surgery	Decayed	3.26	2.50	4.34	3.02	0.05
	Missing	3.34	2.60	4.35	3.12	0.04
	Filled	5.03	4.08	5.72	3.86	0.39
	DMFT	11.64	6.71	14.41	5.88	0.03
	Age	35.26	7.79	37.42	7.12	0.21

**Table 4:** Mean DMFT scores by age group and sex in incarcerated and control groups.

		Control		Prisoner		
Age Group	Sex	n	Mean DMFT	n	Mean DMFT	*p*-Value
<30	F	6	11.3	6	11.7	0.79
<30	M	23	13.4	23	14.1	0.46
30–39	F	10	13.3	10	15.0	0.20
30–39	M	58	10.1	58	14.0	0.01
40+	F	4	9.8	4	17.8	0.01
40+	M	36	12.2	36	15.2	0.06

**Table 5:** Percentage distribution of DMFT components and Dental Treatment Index in incarcerated and control Groups.

Metric	Prisoners	Control	*p*-Value
Decayed (%)	43.8	31.4	0.051
Missing (%)	32.8	30.1	0.570
Filled (%)	30.6	39.0	0.057
TI (%)	63.4	69.1	0.270

**Table 6:** Prevalence of dental treatment needs in incarcerated and control groups.

Metric	Prisoners	Controls	*p*-Value
≥1 Decayed Tooth (%)	94.9	84.6	0.009
≥1 Missing Tooth (%)	95.6	80.1	<0.001
Needing Restoration (%)	94.9	84.6	0.009

## Data Availability

The original contributions presented in this study are included in the article. Further inquiries can be directed to the corresponding author.
